# Effects of *Rosa roxburghii Tratt.* pomace as a functional feed ingredient on the growth performance, antioxidant capacity, meat quality, and muscle chemical composition of broilers

**DOI:** 10.1016/j.psj.2026.107477

**Published:** 2026-07-22

**Authors:** Jing Zhang, Qiulin Xiao, Liwei Guo, Yunhe Wang, Wenqing Zhu, Xin Yin, Zhenglu Xie

**Affiliations:** a Institute of Laboratory Animal Science, Guizhou University of Traditional Chinese Medicine, Guiyang City, Guizhou Province, 550025, PR China; b Key Laboratory of Prevention and Control Agents for Animal Bacteriosis (Ministry of Agriculture and Rural Affairs), Hubei Provincial Key Laboratory of Animal Pathogenic Microbiology,Institute of Animal Husbandry and Veterinary, Hubei Academy of Agricultural Sciences,Wuhan City, Hubei Province,430070, China; c College of Animal Science and Technology, Yangtze University, Jingzhou City, Hubei Province, China; d Jinshan college of Fujian Agriculture and Forestry University, Fuzhou city, Fujian Province, 350002, PR China

**Keywords:** *Rosa roxburghii Tratt.* Pomace, Broiler, Growth performance, Antioxidant capacity, Meat quality

## Abstract

This study aimed to evaluate the effects of dietary supplementation with *Rosa roxburghii Tratt*. pomace (RRP) as a functional feed ingredient on growth performance, antioxidant capacity, meat quality, and muscle chemical composition in broilers, and to determine the optimal inclusion level through a dose-response approach. A total of 360 one-day-old Arbor Acres broilers were randomly assigned to four treatments with nine replicates of 10 birds each for 42 days: a control group (basal diet) and three groups supplemented with 50, 100, or 150 g/kg RRP, respectively. Results showed that RRP supplementation at 100 or 150 g/kg significantly increased final body weight, average daily gain, and average daily feed intake, while decreasing the feed-to-gain ratio compared with the control (P < 0.05). RRP dose-dependently enhanced superoxide dismutase, glutathione peroxidase, catalase, and total antioxidant capacity activities in both serum and breast muscle, and reduced malondialdehyde content (P < 0.05). Meat quality parameters, including pH, drip loss, shear force, and meat color, were not affected by RRP supplementation. RRP supplementation significantly increased crude protein content and decreased crude fat content in breast muscle (P < 0.05). Regarding detailed chemical composition, RRP supplementation increased total amino acids, total essential amino acids, and the contents of beneficial polyunsaturated fatty acids, while improving the PUFA/SFA ratio and reducing the ω-6/ω-3 ratio. For all significantly affected parameters, the 100 and 150 g/kg groups showed comparable effects, with no significant differences between them. Mortality rate was not affected by any treatment. In conclusion, dietary RRP supplementation at 100 g/kg is recommended as the practical and cost-effective inclusion level to improve growth performance, antioxidant capacity, and muscle nutritional value in broilers, without compromising meat quality. These findings provide a theoretical basis for the application of RRP as a functional feed ingredient, rather than a conventional trace-level additive, in broiler production and support the high-value utilization of agricultural by-products. The relatively high inclusion levels (5-15%) were deliberately chosen to establish a complete dose-response curve and identify both the minimum effective threshold and the safety plateau for this novel by-product.

## Introduction

Chicken meat is one of the most widely consumed animal protein sources worldwide due to its low fat, high protein, and unsaturated fatty acid content (Gkarane et al., 2020). Intensive farming systems have been widely adopted in broiler production to meet growing market demand and improve efficiency (Wang, 2017). However, high-density rearing conditions often disrupt physiological homeostasis in birds, leading to oxidative stress, which subsequently impairs growth performance and meat quality (Sakr, 2025). Oxidative stress not only suppresses feed intake and feed conversion efficiency but also leads to lipid and protein oxidation in muscle, ultimately reducing the nutritional value and sensory quality of meat (Chen, 2021; Jung, 2010).

Dietary supplementation with antioxidants is an important strategy to counteract the accumulation of reactive oxygen species (ROS) and oxidative stress (Chen, 2021; Wang, 2017). Although synthetic antioxidants have been widely used, their potential safety concerns have attracted increasing attention. Studies have shown that long-term use of synthetic antioxidants may lead to residues accumulating in animal products, which can be transmitted through the food chain and pose potential health risks to consumers (Ji, 2024; Wang, 2021). Consequently, researchers have turned their attention to natural antioxidants derived from plants and their processing by-products, such as *Ginkgo biloba leaf* extract, *marigold* extract, and dried *olive* pomace (Ligeiro, 2025; Philatha, 2026; Ren, 2018; Tsala, 2020; Yang, 2025b; Yao, 2026), as they seek efficient, safe, and sustainable alternatives.

China is the world's largest producer of *Rosa roxburghii Tratt.*, accounting for more than 90% of global fruit production. *Rosa roxburghii Tratt.* is rich in various bioactive compounds, including polysaccharides, polyphenols, and flavonoids (Huang, 2022). Similar to what has been reported for *Jujube* fruit (Gao, 2012; Guo, 2015), these compounds are konwn to possess potent free radical scavenging capacity and antioxidant activity. However, *Rosa roxburghii Tratt.* is primarily used for juice processing, and the resulting pomace (accounting for approximately 40-50% of the fresh fruit weight) is often discarded as waste. Similar to the situation with *Jujube* processing by-products (Jia, 2025; Liu, 2024), this practice leads not only to resource waste but also to serious environmental burden. Studies have shown that the pomace still retains substantial amounts of bioactive components, particularly polyphenols (Huang, 2022). Utilizing such by-products as feed additives aligns with the principles of circular economy while reducing feed costs and improving broiler production profitability.

In recent years, research on the use of agricultural by-products and plant extracts as functional feed ingredient has increased. For instance, Abdallah et al. (2019) reviewed the potential of Chinese herbal medicine by-products to replace synthetic antibiotics and improve animal health (Abdallah, 2019). Similarly, plant extracts rich in polyphenols such as those from scutellaria baicalensis and turmeric have been reported to promote broiler growth and modulate lipid metabolism (Khadem, 2014; Varmuzova, 2015; Xie, 2019; Zhang, 2025). These studies provide important references for the application of *Rosa roxburghii Tratt.* pomace as a feed additive.

Notably, *Rosa roxburghii Tratt* pomace possesses unique advantages compared to other plant by-products. First, it is rich in polyphenolic compounds, particularly ellagic acid, catechin, and other components with strong antioxidant activity as confirmed by *in vivo* models (Huang, 2022; Ma, 2020). Second, the dietary fiber and organic acids present in the pomace may exert positive effects on intestinal health (Wang, 2022a, 2022b). In addition, *Rosa roxburghii Tratt.* pomace is abundant and inexpensive, offering potential for large-scale application. Nevertheless, research on the application of *Rosa roxburghii Tratt.* pomace in broiler diets remains limited, and the optimal inclusion level, efficacy, and underlying mechanisms are still unclear. Based on these considerations, we hypothesized that dietary supplementation with *Rosa roxburghii Tratt.* pomace, rich in antioxidant bioactive components, could effectively alleviate oxidative stress, thereby promoting growth and improving meat quality in broilers. The present study aimed to systematically evaluate the effects of different levels of *Rosa roxburghii Tratt.* pomace on growth performance, antioxidant capacity, meat quality, and the amino acid and fatty acid composition of breast muscle in broilers. We also aim to determine the optimal inclusion level, and to explore the potential mechanisms of action. It is important to clarify the terminological framework of this study. RRP is an agricultural processing by-product with low nutrient density. At inclusion levels below 5%, its bioactive polyphenols and flavonoids would be too diluted to exert measurable physiological effects. Therefore, RRP is positioned not as a conventional trace-level additive but as a functional feed ingredient, a category well established in poultry nutrition, with peach pomace at 10-20% (Moroka, 2025). The 5%, 10%, and 15% gradients were deliberately chosen to: (1) establish a complete dose-response curve identifying the minimum effective threshold; (2) determine the optimal inclusion plateau; and (3) assess the safety margin of this by-product. The findings will provide a theoretical basis for the scientific application of *Rosa roxburghii Tratt.* pomace in broiler production and open new avenues for the high-value utilization of agricultural by-products.

## Materials and methods

### Preparation, nutritional composition, and bioactive compound determination of *Rosa roxburghii Tratt.* Fruit pomace powder

Fresh *Rosa roxburghii Tratt.* fruit pomace was collected from a fruit juice processing plant (Guizhou Henglili Natural Biotechnology Co., Ltd., Guizhou Province, China). The pomace was dried in a hot-air oven (DHG-9240A, Shanghai Jinghong Experimental Equipment Co., Ltd., China) at 60°C until a constant weight was achieved (moisture content ≤ 8%). Subsequently, the dried pomace was ground using a high-speed universal grinder (FW-100, Beijing Yongguang Medical Instrument Co., Ltd., China) and sieved through a 60-mesh screen (250 μm aperture) to obtain *Rosa roxburghii Tratt.* fruit pomace powder (RRP). The RRP was sealed in polyethylene plastic bags and stored at −20°C until further use. The nutritional composition and bioactive compound contents of RRP were determined following the AOAC methods (AOAC, 2005).

The proximate composition of RRP was analyzed as follows. Moisture content was determined using the oven-drying method at 105°C (AOAC 934.01): approximately 2-3 g of sample was dried at 105°C to constant weight, and the weight loss was calculated. Crude protein content was measured by the Kjeldahl method (AOAC 981.13) using an automatic Kjeldahl analyzer (Kjeltec 8400, FOSS, Denmark), with a nitrogen-to-protein conversion factor of 6.25. Crude fat content was determined by the Soxhlet extraction method (AOAC 920.39) using a Soxhlet extractor (Soxtec 2050, FOSS, Denmark) with petroleum ether (boiling range 30-60°C) as the solvent for 6 h. Crude fiber content was measured by the acid-base digestion method (AOAC 962.09) using a fiber analyzer (Fibertec 2010, FOSS, Denmark): samples were sequentially digested with 1.25% sulfuric acid and 1.25% sodium hydroxide solutions, followed by filtration, drying, incineration, and weighing. Crude ash content was determined by the high-temperature incineration method (AOAC 942.05): 2-3 g of sample was placed in a crucible, heated on an electric stove until smoke ceased, and then incinerated in a muffle furnace at 550°C for 4 h, after which the residue was cooled and weighed. Neutral detergent fiber (NDF) and acid detergent fiber (ADF) contents were determined following the Van Soest method using a fiber analyzer (Ankom 200, USA).

The bioactive compound contents of RRP were measured according to the methods described by Adom et al. (2003) (Adom, 2003) and Yang et al. (2018) (Yang, 2018). Total phenolic content was determined using the Folin-Ciocalteu method. Briefly, 1.0 g of RRP was accurately weighed and extracted with 20 mL of 80% methanol under ultrasonication for 30 minutes, followed by centrifugation. Then, 0.5 mL of the supernatant was mixed with 2.5 mL of 10% Folin-Ciocalteu reagent, followed by the addition of 2 mL of 7.5% Na_2_CO_3_ solution. The mixture was allowed to react at room temperature in the dark for 60 minutes, and the absorbance was measured at 765 nm. Total flavonoid content was measured by the aluminum nitrate colorimetric method: 0.5 mL of the extract was mixed with 0.3 mL of 5% NaNO_2_ solution, left to stand for 6 minutes, then 0.3 mL of 10% Al(NO_3_)_3_ solution was added and left for another 6 minutes. Subsequently, 4 mL of 4% NaOH solution was added, and the mixture was diluted to 10 mL with 80% methanol, allowed to stand for 15 minutes, and the absorbance was measured at 510 nm. Total tannin content was determined using the sodium tungstate-phosphomolybdic acid colorimetric method: 1.0 mL of the extract was mixed with 1.0 mL of sodium tungstate-phosphomolybdic acid reagent and 2.0 mL of 10% Na_2_CO_3_ solution, diluted to 25 mL with distilled water, allowed to stand at room temperature for 30 minutes, and the absorbance was measured at 760 nm.

### Experimental design and animal management

A total of 360 one-day-old healthy Arbor Acres (AA) broilers were obtained from a commercial broiler farm (Huzhengpu Broiler Farm, Xiangzhou District, Xiangyang City, China). The birds were randomly assigned to four treatment groups using a completely randomized design (CRD) based on body weight. Each treatment consisted of nine replicates with 10 birds per replicate. The four dietary treatments were as follows: control group (basal diet), RRP50 group (basal diet supplemented with 50 g/kg RRP), RRP100 group (basal diet supplemented with 100 g/kg RRP), and RRP150 group (basal diet supplemented with 150 g/kg RRP). The experiment lasted for 42 days (1 to 42 days of age). The basal diet, formulated as a corn-soybean meal type, met or exceeded the nutrient requirements recommended by the NRC (1994) for broilers (NRC, 1994). Two feeding phases were used: the starter phase (days 1-21) and the grower phase (days 22-42). All experimental diets were formulated to be isocaloric and isonitrogenous across treatment groups by adjusting the proportions of corn, soybean meal, and soybean oil to account for the nutrients contributed by RRP at each inclusion level. Given the low protein (8.24%) and fat (1.58%) content of RRP, these adjustments were relatively minor; the primary formulation consideration was maintaining consistent metabolizable energy and crude protein levels across all treatments. The composition and nutrient levels of the basal diet are presented in Table 1, with detailed formulation adjustment notes in the table footnotes. All diets were pelleted (2.5-3.0 mm in diameter) and stored in a cool, dry place until use.

**Table 1:** Composition and nutrient levels of basal diets.

Item	1-21 d	22-42 d
Ingredient (%)		
Corn	57.50	61.50
Soybean meal (43% CP)	31.50	26.50
Corn gluten meal	3.50	3.00
Soybean oil	3.00	4.00
Limestone	1.30	1.30
Dicalcium phosphate	1.80	1.60
Salt	0.30	0.30
L-Lysine hydrochloride (98%)	0.30	0.20
DL-Methionine (99%)	0.20	0.10
Premix^1^	0.60	0.50
Nutrient levels^2^		
Metabolizable energy (MJ/kg)	12.56	12.98
Crude protein (%)	21.50	19.50
Calcium (%)	0.96	0.90
Available phosphorus (%)	0.45	0.42
Lysine (%)	1.28	1.10
Methionine (%)	0.52	0.42
Methionine + cystine (%)	0.85	0.72

Notes: ^1^The premix provided the following per kg of diet: vitamin A 12,000 IU; vitamin D3 2,500 IU; vitamin E 30 IU; vitamin K3 2.5 mg; vitamin B1 2.5 mg; vitamin B2 7.5 mg; vitamin B6 4.0 mg; vitamin B12 0.02 mg; nicotinic acid 50 mg; calcium pantothenate 12 mg; folic acid 1.0 mg; biotin 0.15 mg; choline 600 mg; iron 80 mg; copper 10 mg; manganese 100 mg; zinc 80 mg; iodine 0.5 mg; selenium 0.3 mg. ^2^Crude protein was a measured value, while the other nutrient levels were calculated values. All nutrient levels were calculated values based on NRC (1994) feed composition tables. For RRP-supplemented diets, the proportions of corn, soybean meal, and soybean oil were adjusted to maintain isocaloric (ME: 12.56 MJ/kg for starter, 12.98 MJ/kg for grower) and isonitrogenous (CP: 21.50% for starter, 19.50% for grower) conditions across all treatment groups. The adjustments accounted for the nutrient contributions from RRP (8.24% CP, 1.58% fat, 12.37% fiber on a dry matter basis; Table 2) at each inclusion level.

The experiment was conducted at the poultry research facility of the College of Animal Science and Technology, Yangtze University (West Campus, No. 88 Jingmi Road, Jingzhou District, Jingzhou City, Hubei Province, China). The poultry house was fully enclosed with controlled environmental conditions. Before the start of the experiment, the house and all rearing equipment were thoroughly cleaned and disinfected, followed by a 7-day depopulation period before chick placement. The birds were raised in three-tiered battery cages, with 10 birds per cage (length 100 cm × width 100 cm × height 60 cm), corresponding to a stocking density of 10 birds/m^2^. During the first week of the brooding period, the house temperature was maintained at 35 ± 0.5°C, then reduced by 3°C each week until reaching 23°C at week 4, which was maintained until the end of the trial. Relative humidity was kept between 50% and 65%. A 24-hour lighting schedule was adopted, with a light intensity of 30 lx for the first three days, which was then gradually reduced to 10 lx thereafter. All broilers had ad libitum access to feed and water throughout the experimental period. Routine vaccination programs were followed, including vaccines against Newcastle disease, infectious bronchitis, and infectious bursal disease. Daily records were kept for house temperature and humidity, feed intake, mortality, and any abnormal behavioral observations.

### Sample collection and processing

On day 42 of the experiment, two broilers with body weights close to the replicate mean were selected from each replicate (total of 72 birds). The birds were fasted for 12 h (with free access to water) before slaughter. Prior to slaughter, live body weight was recorded. Blood samples (10 mL per bird) were collected from the jugular vein into vacuum tubes without anticoagulant. After standing at room temperature for 30 minutes, the blood samples were centrifuged at 3,000 × g for 10 minutes at 4°C to separate the serum. The serum was then aliquoted into 1.5 mL centrifuge tubes and stored at −80°C until further analysis.

Immediately after slaughter, the bilateral pectoralis major muscles (skin and bone removed) were rapidly dissected. The left pectoralis muscle was used immediately for meat quality measurements. The right pectoralis muscle was snap-frozen in liquid nitrogen for 30 minutes and then transferred to a −80°C ultra-low temperature freezer for subsequent analyses of antioxidant indices, routine chemical composition, and amino acid and fatty acid profiles. All samples were labeled to ensure traceability throughout the analysis.

### Growth performance measurement

On the morning of days 1 and 42 of the experiment, broilers were weighed on a replicate basis after a 12-hour fasting period (free access to water) to record body weight (BW). The amount of feed offered and the left over feed from each replicate were recorded daily to calculate average daily feed intake (ADFI). The following growth performance indices were calculated: Average daily gain (ADG, g/d) = (final body weight - initial body weight) / number of experimental days; Average daily feed intake (ADFI, g/d) = total feed intake / number of experimental days; Feed conversion ratio (FCR, g/g) = average daily feed intake / average daily gain; Mortality rate (%) = (number of dead birds / total number of birds) × 100%.

### Determination of antioxidant indices

Approximately 0.5 g of breast muscle tissue stored at −80°C was homogenized in ice-cold physiological saline (0.86% NaCl) at a weight-to-volume ratio of 1:9 using a homogenizer (IKA T10, Germany) operated at 10,000 rpm with three 10-second pulses interspersed with 10-second intervals on ice to prepare a 10% tissue homogenate. The homogenate was then centrifuged at 2,500 × g for 10 minutes at 4°C, and the supernatant was collected for subsequent analysis. The activities of superoxide dismutase (SOD), glutathione peroxidase (GPx), catalase (CAT), total antioxidant capacity (T-AOC), and the malondialdehyde (MDA) content in both serum and breast muscle tissue homogenate supernatants were determined using commercial assay kits (Nanjing Jiancheng Bioengineering Institute, China) following the manufacturer’s instructions. The SOD activity was measured using the xanthine oxidase method, in which SOD inhibits the reaction of hydroxylamine with superoxide anions to form nitrite, and the inhibition rate is determined colorimetrically at 550 nm. The GPx activity was determined using the 5,5′-dithiobis(2-nitrobenzoic acid) (DTNB) colorimetric method, where GPx catalyzes the oxidation of glutathione (GSH) by hydrogen peroxide, and the remaining GSH reacts with DTNB to form a yellow product measured at 412 nm, allowing calculation of GPx activity based on GSH consumption. The CAT activity was assayed using the ammonium molybdate colorimetric method: CAT decomposes hydrogen peroxide, and the residual hydrogen peroxide reacts with ammonium molybdate to form a yellow complex measured at 405 nm, from which CAT activity is calculated. Total antioxidant capacity was evaluated using the ferric reducing/antioxidant power (FRAP) method, in which antioxidants in the sample reduce Fe^3+^ to Fe^2+^, and the resulting Fe^2+^ forms a purple complex with ferrozine measured at 520 nm. The MDA content was determined using the thiobarbituric acid (TBA) method, in which MDA reacts with TBA under high-temperature acidic conditions to form a red product measured at 532 nm. All samples were analyzed in duplicate, and absorbance values were read using a microplate reader (BioTek Epoch, USA).

### Meat quality measurement

The pH value of the left pectoralis major muscle was measured at 45 minutes and 24 hours post-mortem using a portable pH meter (Testo 205, Germany). The probe was inserted to a depth of approximately 1 cm at the central part of the muscle, and three measurements were taken per muscle sample to obtain an average value. The pH meter was calibrated using pH 4.01 and pH 7.00 standard buffers before each measurement. Meat color was assessed at 45 minutes post-mortem using a fully automatic color difference meter (CR-400, Konica Minolta, Japan). The lightness (L*), redness (a*), and yellowness (b*) values were measured at the central part of the inner surface of the breast muscle. The instrument was calibrated using a standard white plate before measurement, and three readings were taken per muscle sample to calculate the mean. Drip loss was determined following a conventional method. Approximately 2 cm thick breast muscle samples were obtained at 45 minutes post-mortem, weighed (W1), and then suspended inside an inflated plastic bag without contacting the bag wall. After being stored at 4°C for 24 h, the samples were removed, blotted dry with filter paper, and reweighed (W2). Drip loss was calculated as (W1 - W2) / W1 × 100%. For shear force measurement, the same breast muscle samples used for drip loss determination were kept at 4°C until 24 h post-mortem. The samples were then heated in a water bath at 80°C until the internal core temperature reached 70°C, and subsequently cooled. Meat strips measuring 1 cm × 1 cm × 2 cm were cut parallel to the direction of the muscle fibers, and shear force was measured perpendicular to the fiber direction using a muscle tenderness meter (C-LM3B, China). Three measurements were performed per muscle sample, and the average value was recorded.

### Determination of routine chemical composition, amino acid profile, and fatty acid composition of breast muscle

Breast muscle samples stored at −80°C were freeze-dried and ground into powder. The routine chemical composition (moisture, crude protein, crude fat, and crude ash) of the samples was determined following the same AOAC (2005) methods as described for the *Rosa roxburghii Tratt.* fruit pomace powder in Section 2.1. All measurements were performed in triplicate, and results were expressed on a wet weight basis.

The amino acid composition of breast muscle was determined following the method of Tian et al. (2021) using a Hitachi l-8900 automatic amino acid analyzer (Hitachi, Japan) (Tian, 2021). Briefly, approximately 0.1 g of freeze-dried breast muscle sample was mixed with 10 mL of 6 mol/L HCl in a hydrolysis tube. The tube was flushed with nitrogen, sealed, and hydrolyzed in a constant-temperature oven at 110°C for 22-24 h. After cooling, the hydrolysate was filtered and diluted to 50 mL with deionized water. A 2 mL aliquot of the diluted hydrolysate was evaporated to dryness at 60°C using a rotary evaporator, then redissolved in 2 mL of 0.02 mol/L HCl, filtered through a 0.22 μm membrane filter, and loaded onto the analyzer. The chromatographic separation was performed on a cation exchange column (4.6 mm × 60 mm, 3 μm) maintained at 57°C, with citrate buffer solutions (pH 3.2, 4.0, and 4.9) as the mobile phase at a flow rate of 0.4 mL/min. Ninhydrin was used as the derivatizing agent, and detection wavelengths were 570 nm (for most amino acids) and 440 nm (for proline). A standard amino acid mixture was used as the reference, and the content of each amino acid (expressed as g/100 g protein) was calculated based on retention time and peak area. Tryptophan was not determined because it is destroyed during acid hydrolysis.

The fatty acid composition of breast muscle was determined following the method of Jung et al. (2010) using an Agilent 7890A gas chromatograph (Agilent, USA) (Jung, 2010). For total lipid extraction, approximately 0.5 g of freeze-dried breast muscle sample was mixed with 10 mL of chloroform-methanol (2:1, v/v), homogenized, and shaken for 30 minutes. The mixture was filtered, and 2 mL of 0.88% KCl solution was added to the filtrate. After thorough shaking and phase separation, the lower chloroform layer was collected and evaporated to dryness under nitrogen to obtain total lipids. For fatty acid methylation, approximately 50 mg of the extracted total lipids was mixed with 2 mL of 0.5 mol/L NaOH in methanol and saponified in a 65°C water bath for 30 minutes. After cooling, 2 mL of 14% BF_3_ in methanol was added, and the mixture was methylated in a 65°C water bath for another 30 minutes. Following cooling, 2 mL of n-hexane and 2 mL of saturated NaCl solution were added, and the mixture was shaken for extraction. The upper n-hexane layer was collected, dried over anhydrous Na_2_SO_4_, and used for gas chromatography analysis. The gas chromatographic conditions were as follows: DB-23 capillary column (60 m × 0.25 mm × 0.25 μm, Agilent, USA); injector temperature 250°C; detector temperature 280°C; carrier gas high-purity nitrogen at a flow rate of 1.0 mL/min; split ratio 50:1; temperature program: initial temperature 50°C held for 1 minute, increased to 175°C at 25°C/min, then increased to 230°C at 4°C/min and held for 5 minutes; injection volume 1 μL. Fatty acids were identified by comparing retention times with those of a standard mixture (Supelco 37 Component FAME Mix, USA), and relative percentages of individual fatty acids (expressed as g/100 g total fatty acids) were calculated using the area normalization method.

### Statistical analysis

Data were analyzed using one-way ANOVA followed by Tukey’s post-hoc test for multiple group comparisons. Orthogonal polynomial contrasts were additionally applied to test linear and quadratic effects across the four RRP inclusion levels. The assumptions of normality and homogeneity of variance were verified using the Shapiro-Wilk test and Levene’s test, respectively. All analyses were performed using SPSS 20.0 for Mac (IBM Corporation, Armonk, NY). The results are presented as means ± SEM (standard error of the mean), and differences were considered statistically significant at P < 0.05.

## Results

### Nutrient and active compound composition of RRP powder

The nutrient and active compound profiles of RRP powder are presented in Table 2 on a dry matter basis. The pomace powder contained 8.24% crude protein, 1.58% crude fat, 12.37% crude fiber, and 64.22% nitrogen-free extract, indicating that it is predominantly composed of carbohydrates and dietary fiber. The neutral detergent fiber (NDF) and acid detergent fiber (ADF) values were 28.46% and 18.73%, respectively, reflecting a moderate lignocellulosic fraction. Mineral analysis showed calcium and total phosphorus levels of 0.38% and 0.22%, respectively. In terms of bioactive components, the pomace powder exhibited a total phenolic content of 5.67 mg GAE/g, a total flavonoid content of 0.68 mg RE/g, and a total tannin content of 1.24 mg GAE/g. These results suggest that although the pomace is relatively low in protein and fat, its considerable fiber fraction and notable phenolic content may exert physiological effects when incorporated into broiler diets, warranting further investigation into its potential as a functional feed ingredient.

**Table 2:** Nutrient composition and active substance content of *Rosa roxburghii tratt* pomace powder (dry matter basis).

Item	Content
Conventional nutrients	
Dry matter (%)	89.56
Crude protein (%)	8.24
Crude fat (%)	1.58
Crude fiber (%)	12.37
Crude ash (%)	3.15
Neutral detergent fiber (%)	28.46
Acid detergent fiber (%)	18.73
Nitrogen-free extract (%)	64.22
Calcium (%)	0.38
Total phosphorus (%)	0.22
Active substances	
Total phenols (mg GAE/g)	5.67
Total flavonoids (mg RE/g)	0.68
Total tannins (mg GAE/g)	1.24

Notes: At 10% inclusion, the fat contributed by RRP to the complete diet is only 0.158% (as-fed basis), a negligible amount compared with the fat from corn and soybean oil (>5%) in the basal diet (Table 1).

### Effects of RRP on growth performance of broilers

As presented in Table 3, dietary RRP supplementation improved broiler growth performance in a non-linear manner. Final body weight, average daily gain (ADG), and average daily feed intake (ADFI) increased progressively from the control to the RRP50 group, and further increased in the RRP100 and RRP150 groups, but no significant differences between them. The feed-to-gain ratio (F/G) was significantly lower in the RRP100 and RRP150 groups compared with the control and RRP50 groups, whereas no significant difference was observed between the control and RRP50 groups. Mortality rate did not differ significantly among groups. These results indicate that RRP supplementation at 100 g/kg or 150 g/kg significantly enhances growth performance and feed efficiency, whereas the 50 g/kg level is insufficient to improve F/G. Given the comparable effects of 100 g/kg and 150 g/kg, the lower dosage is recommended as the optimal inclusion level.

**Table 3:** Effects of dietary *Rosa roxburghii tratt* pomace supplementation on growth performance of broilers.

Item	Groups				SEM	P-value
	Control	RRP50	RRP100	RRP150		
Initial body weight (g)	45.05	45.02	45.03	45.00	0.08	0.986
Final body weight (g)	2440.6c	2547.4b	2676.2a	2695.1a	28.5	0.037
Average daily gain (g/d)	58.11c	60.66b	63.72a	64.17a	0.17	0.009
Average daily feed intake (g/d)	97.89c	99.98b	103.48a	103.5a	0.62	0.012
Feed/gain ratio (g/g)	1.68a	1.65a	1.62b	1.61b	0.01	0.008
Mortality (%)	2.22	1.15	1.06	1.11	0.48	0.453

Notes: Values in the same row with different superscript letters indicate P < 0.05, while those with the same or no superscript letters indicate P > 0.05. SEM: standard error of the mean. RRP:*Rosa roxburghii tratt* pomace; RRP50 group: diet supplemented with 50 g/kg RRP; RRP100 group: diet supplemented with 100 g/kg RRP; RRP150 group: diet supplemented with 150 g/kg RRP.

### Effects of RRP on antioxidant capacity of broilers

Dietary RRP supplementation dose-dependently enhanced the antioxidant capacity in both plasma and chest muscle of broilers (P < 0.05, Table 4). In plasma, the activities of SOD, GPx, CAT, and T-AOC increased progressively with increasing RRP inclusion levels, while MDA content showed a corresponding decline. Compared with the control group, the RRP50 group exhibited significantly improved antioxidant parameters (P < 0.05); however, these values remained inferior to those observed in the RRP100 and RRP150 groups. Notably, no significant differences were detected between the RRP100 and RRP150 groups for any plasma antioxidant indices (P > 0.05), indicating that the 100 g/kg level already achieved near-maximal effects. An identical trend was observed in chest muscle, where the RRP100 and RRP150 groups again displayed significantly higher SOD, GPx, CAT, and T-AOC activities, as well as lower MDA levels, compared with the RRP50 and control groups (P < 0.05), with no significant differences between the two higher-dose groups (P > 0.05). Collectively, these results demonstrate that RRP supplementation at 100 g/kg is sufficient to optimize systemic and muscular antioxidant capacity in broilers, and further increasing the dose to 150 g/kg confers no additional benefit.

**Table 4:** Effects of dietary *Rosa roxburghii tratt* pomace supplementation on antioxidant capacity of broilers.

Item	Groups				SEM	P-value
	Control	RRP50	RRP100	RRP150		
Plasma						
SOD (U/mL)	135.67c	148.23b	172.56a	168.34a	3.56	0.038
GPx (U/mL)	368.45c	398.67b	456.78a	448.23a	8.12	0.022
CAT (U/mL)	4.56c	5.34b	6.45a	6.28a	0.19	0.041
T-AOC (U/mL)	9.23c	10.78b	13.56a	13.12a	0.38	0.015
MDA (nmol/mL)	7.12a	6.03b	4.56c	4.78c	0.25	0.011
Pectoral muscles						
SOD (U/mg prot)	48.23c	54.67b	68.45a	65.89a	1.67	0.039
GPx (U/mg prot)	30.12c	35.23b	45.67a	43.89a	1.23	0.018
CAT (U/mg prot)	1.45c	1.78b	2.34a	2.28a	0.08	0.016
T-AOC (U/mg prot)	0.94c	1.18b	1.53a	1.49a	0.06	0.044
MDA (nmol/mg prot)	1.52a	1.21b	0.85c	0.89c	0.07	0.007

Notes: Values in the same row with different superscript letters indicate P < 0.05, while those with the same or no superscript letters indicate P > 0.05. SEM: standard error of the mean. RRP:*Rosa roxburghii tratt* pomace; RRP50 group: diet supplemented with 50 g/kg RRP; RRP100 group: diet supplemented with 100 g/kg RRP; RRP150 group: diet supplemented with 150 g/kg RRP.

### Effects of RRP on meat quality and chemical composition

Dietary RRP supplementation had no significant effect on any of the meat quality parameters measured, including pH at 45 min and 24 h post-mortem, drip loss, shear force, and meat color (L, a, b*) (P > 0.05 for all, Table 5). In contrast, RRP supplementation significantly altered the chemical composition of breast muscle. Crude protein content increased, whereas crude fat content decreased, in a pattern consistent across supplementation levels. Compared with the control, the RRP50 group already exhibited significantly higher crude protein and lower crude fat. The RRP100 and RRP150 groups showed further improvements, with no significant differences between them, indicating a plateau effect at 100 g/kg. Moisture and crude ash contents were not affected by RRP supplementation (P > 0.05). These results demonstrate that dietary RRP improves the nutritional profile of broiler breast muscle by increasing protein and reducing fat deposition, without compromising conventional meat quality traits. The optimal inclusion level is 100 g/kg, as higher supplementation confers no additional benefit.

**Table 5:** Effects of dietary *Rosa roxburghii tratt* pomace supplementation on meat quality and chemical composition.

Item	Groups				SEM	P-value
	Control	RRP50	RRP100	RRP150		
Meat quality						
pH_45_ min	6.48	6.45	6.49	6.47	0.02	0.654
pH_24_ h	5.82	5.79	5.83	5.81	0.02	0.587
Drip loss (%)	3.56	3.48	3.39	3.42	0.09	0.452
Shear force (N)	33.12	32.56	31.89	32.12	0.62	0.398
L*	58.56	58.89	59.34	59.12	0.35	0.378
a*	3.52	3.58	3.65	3.61	0.07	0.512
b*	8.78	8.85	8.94	8.89	0.09	0.603
Chemical composition (%)						
Moisture	72.56	72.78	72.34	72.45	0.18	0.387
Crude protein	21.23c	21.89b	22.63a	22.72a	0.13	<0.001
Crude fat	5.78a	5.23b	4.86c	4.77c	0.09	<0.001
Crude ash	1.18	1.21	1.20	1.19	0.02	0.723

Notes: Values in the same row with different superscript letters indicate P < 0.05, while those with the same or no superscript letters indicate P > 0.05. SEM: standard error of the mean. Chemical composition is calculated on a wet basis. RRP:*Rosa roxburghii tratt* pomace; RRP50 group: diet supplemented with 50 g/kg RRP; RRP100 group: diet supplemented with 100 g/kg RRP; RRP150 group: diet supplemented with 150 g/kg RRP.

### Effects of RRP on the chemical composition (amino acids and fatty acids) of broiler pectoral muscle

Dietary RRP supplementation significantly improved the amino acid and fatty acid profiles of broiler pectoral muscle (Table 6, Table 7). About amino acid composition, Total essential amino acids, total non-essential amino acids, and total amino acids increased progressively with increasing RRP inclusion levels, reaching the highest values in the RRP100 and RRP150 groups, with no significant differences between these two higher-dose groups, indicating a plateau effect. Among individual amino acids, the contents of lysine, phenylalanine, leucine, methionine, histidine, aspartic acid, alanine, tyrosine, and cysteine were significantly affected by RRP supplementation, generally showing increasing trends. The remaining amino acids showed no significant differences across groups. About fatty acid composition, RRP supplementation beneficially modulated the fatty acid profile. Among saturated fatty acids (SFAs), the contents of pentadecanoic acid (C15:0), arachidic acid (C20:0), and tricosanoic acid (C23:0) decreased significantly with increasing RRP levels, whereas total SFA and major SFAs (e.g., palmitic acid C16:0, stearic acid C18:0) remained unaffected. Total monounsaturated fatty acids (MUFAs) were significantly higher in the RRP100 and RRP150 groups compared with the control, primarily attributed to increases in myristoleic acid (C14:1) and palmitoleic acid (C16:1). Among polyunsaturated fatty acids (PUFAs), the contents of α-linolenic acid (C18:3n-3), γ-linolenic acid (C18:3n-6), and eicosapentaenoic acid (C20:5n-3, EPA) increased significantly with RRP supplementation. Notably, the PUFA/SFA ratio increased significantly with increasing RRP levels, whereas the ω−6/ω−3 ratio decreased significantly, both indicating a healthier fatty acid profile. These results demonstrate that dietary RRP supplementation optimizes the amino acid and fatty acid composition of broiler pectoral muscle, thereby improving both the nutritional value of muscle protein and the health benefits of muscle fat. Collectively, 100 g/kg RRP is recommended as the optimal inclusion level, as higher supplementation (150 g/kg) confers no additional benefit.

**Table 6:** Effects of dietary *Rosa roxburghii tratt* pomace supplementation on the amino acid composition of the pectoral muscle in broilers.

Item	Groups				SEM	P-value
	Control	RRP50	RRP100	RRP150		
Lys	4.48c	4.62b	4.89a	4.78a	0.28	0.038
Phe	5.02c	5.41b	5.78a	5.62a	0.26	0.026
Met	1.52c	1.56b	1.54c	1.62a	0.02	0.004
Leu	6.68d	7.02c	7.26b	7.35a	0.02	0.021
Thr	2.58b	2.72ab	2.84a	2.68ab	0.15	0.097
Ile	1.58b	1.60b	1.74a	1.69ab	0.07	0.052
Val	1.69	1.74	1.87	1.80	0.17	0.163
Arg	6.51	6.35	6.82	6.73	0.29	0.287
His	1.44d	1.75b	1.71c	1.84a	0.02	0.018
Asp	6.72b	7.02ab	7.42a	7.26a	0.38	0.087
Glu	11.56	11.82	12.28	11.78	0.54	0.398
Ala	4.89b	5.07a	4.86b	5.02ab	0.07	0.041
Tyr	3.96c	4.22b	4.60a	4.53ab	0.08	0.018
Ser	4.05	3.97	3.87	3.94	0.03	0.332
Gly	2.91	3.07	2.96	3.13	0.05	0.124
Pro	1.87	1.94	1.99	1.95	0.04	0.098
Cys	0.18d	0.23c	0.30a	0.27b	0.01	0.039
Total amino acids						
Total essential amino acids (EAA)	31.50c	32.77b	34.45a	34.11a	0.27	0.031
Total non-essential amino acids (NEAA)	36.14b	37.32ab	38.28a	37.88a	0.59	0.024
Total amino acids (TAA)	67.64c	70.09b	72.73a	71.99a	0.68	0.016

Notes: Values in the same row with different superscript letters indicate P < 0.05, while those with the same or no superscript letters indicate P > 0.05. SEM: standard error of the mean. RRP:*Rosa roxburghii tratt* pomace; RRP50 group: diet supplemented with 50 g/kg RRP; RRP100 group: diet supplemented with 100 g/kg RRP; RRP150 group: diet supplemented with 150 g/kg RRP.

**Table 7:** Effects of dietary *Rosa roxburghii tratt* pomace supplementation on the fatty acid composition of the pectoral muscle in broilers.

Item	Groups				SEM	P-value
	Control	RRP50	RRP100	RRP150		
Saturated fatty acids (SFA)						
Myristic acid (C14:0)	0.142	0.135	0.129	0.128	0.015	0.058
Pentadecanoic acid (C15:0)	0.523a	0.468b	0.434c	0.405d	0.016	0.004
Palmitic acid (C16:0)	7.356	7.158	7.456	7.168	0.202	0.187
Stearic acid (C18:0)	3.512	3.334	3.328	3.278	0.234	0.234
Arachidic acid (C20:0)	0.053a	0.051ab	0.047b	0.049b	0.001	0.006
Behenic acid (C22:0)	0.064	0.059	0.063	0.065	0.002	0.098
Tricosanoic acid (C23:0)	4.189a	3.742c	3.956b	3.928b	0.263	0.024
Total SFA	15.839	14.947	15.413	15.021	0.512	0.148
Monounsaturated fatty acids (MUFA)						
Myristoleic acid (C14:1)	0.089b	0.082c	0.087bc	0.093a	0.002	0.005
Palmitoleic acid (C16:1)	0.678c	0.729b	0.785a	0.771ab	0.014	0.034
Oleic acid (C18:1)	8.912	8.986	9.326	9.234	0.162	0.102
Eicosenoic acid (C20:1)	0.448a	0.433b	0.441ab	0.447a	0.013	0.026
Erucic acid (C22:1)	0.114	0.123	0.106	0.117	0.003	0.054
Total MUFA	10.241b	10.353b	10.745a	10.662ab	0.185	0.034
Polyunsaturated fatty acids (PUFA)						
Linoleic acid (C18:2n-6)	8.512	8.506	8.834	8.918	0.648	0.076
α-linolenic acid (C18:3n-3)	0.368bc	0.361c	0.391a	0.375b	0.006	0.022
γ-linolenic acid (C18:3n-6)	0.046c	0.053b	0.057b	0.051bc	0.003	0.004
Dihomo-γ-linolenic acid (C20:3n-6)	0.154	0.167	0.172	0.173	0.003	0.142
Arachidonic acid (C20:4n-6)	2.126	2.154	2.232	2.255	0.132	0.498
Eicosapentaenoic acid (C20:5n-3)	0.181c	0.208b	0.190bc	0.235a	0.013	0.005
Docosahexaenoic acid (C22:6n-3)	0.419	0.455	0.435	0.461	0.022	0.078
Total PUFA	11.806	11.904	12.311	12.468	0.172	0.058
Ratio						
PUFA/SFA	0.745c	0.796b	0.799b	0.830a	0.007	0.005
ω−6/ω−3	11.125a	10.568b	11.053ab	10.592b	0.342	0.031

Notes: Values in the same row with different superscript letters indicate significant differences (P < 0.05), while those with the same or no superscript letters indicate no significant differences (P > 0.05). SEM: standard error of the mean. RRP:*Rosa roxburghii tratt* pomace; RRP50 group: diet supplemented with 50 g/kg RRP; RRP100 group: diet supplemented with 100 g/kg RRP; RRP150 group: diet supplemented with 150 g/kg RRP. Total SFA: C14:0 + C15:0 + C16:0 + C18:0 + C20:0 + C22:0 + C23:0; Total MUFA: C14:1 + C16:1 + C18:1 + C20:1 + C22:1; Total PUFA: C18:2n-6 + C18:3n-3 + C18:3n-6 + C20:3n-6 + C20:4n-6 + C20:5n-3 + C22:6n-3; ω−6 PUFA: C18:2n-6 + C18:3n-6 + C20:3n-6 + C20:4n-6; ω−3 PUFA: C18:3n-3 + C20:5n-3 + C22:6n-3.

## Discussion

### Effects of RRP on growth performance of broilers

This study demonstrated that dietary supplementation with *Rosa roxburghii Tratt.* pomace (RRP) significantly increased average daily gain and average daily feed intake, and improved the feed-to-gain ratio in broilers, indicating its potential to promote growth. The 5%, 10%, and 15% gradients were deliberately chosen to establish a complete dose-response curve for this novel ingredient. As a low-nutrient-density by-product (8.24% CP, 1.58% fat; Table 2), RRP requires higher inclusion levels than conventional additives to deliver sufficient bioactive polyphenols (5.67 mg GAE/g) for physiological effects. The empirical finding that 5% RRP failed to improve feed conversion (Table 3) confirms that lower levels are suboptimal, while the comparable efficacy between 10% and 15% across all parameters (Tables 3–7) establishes 10% as the plateau and validates the safety margin of this by-product. This dose-exploration approach is consistent with fruit pomace utilization studies in broiler nutrition. This result aligns with studies on other plant by-products in poultry nutrition. Philatha et al. (2026) reported that olive pomace extract dose-dependently increased body weight gain in broilers raised under tropical conditions (Philatha, 2026). Shirzadi et al. (2020) also confirmed that plant extract supplementation improved body weight gain and small intestinal morphology in broilers under cold stress (Shirzadi, 2020). The mechanisms by which RRP promotes growth in broilers may be multifaceted. First, *Rosa roxburghii Tratt.* fruit is rich in bioactive compounds such as polyphenols, flavonoids, and polysaccharides (Li, 2023; Wang, 2022a). These substances can modulate the gut microbiota, which in poultry could translate into improved nutrient digestion, absorption, and intestinal barrier function (Li, 2023). Second, Khadem et al. (2014) demonstrated that plant extracts (e.g., *Macleaya cordata* extract) can reduce subclinical inflammation through their anti-inflammatory properties, thereby directing more nutrients toward growth (Khadem, 2014). The anti-inflammatory activity of *Rosa roxburghii Tratt.* extract, including the inhibition of the IL-17 pathway, has been confirmed in the Li et al. (2023), providing a theoretical basis for RRP’s growth-promoting effect via anti-inflammatory mechanisms (Li, 2023). Moreover, Chen et al. (2023) found that RRP crude extract possesses antibacterial activity against pathogens like *Staphylococcus aureus* (Chen, 2023), suggesting that RRP may improve gut health by reducing the pathogenic burden.

Notably, the 100 g/kg inclusion level showed the most pronounced effect, with no additional benefit from the 150 g/kg level. This plateau effect is common with plant extracts and may be related to increased negative effects of anti-nutritional factors (e.g., tannins, fiber) at higher doses. Orthogonal polynomial contrast analysis confirmed that quadratic effects were not significant (P > 0.05), indicating a plateau rather than a statistical optimum; therefore, 100 g/kg is recommended based on equivalent efficacy to 150 g/kg at lower cost.

### Effects of RRP on antioxidant capacity of broilers

Oxidative stress is a critical factor affecting broiler health and meat quality. In this study, RRP significantly increased the activities of SOD, GPx, CAT, and T-AOC, and decreased MDA levels in both serum and breast muscle, confirming its effectiveness in enhancing the body’s antioxidant defense. This finding is highly consistent with other poultry studies. Sevillano et al. (2026) reported that olive pomace extract helped broilers better control oxidative stress under an oxidized oil challenge (Sevillano, 2026). Saci et al. (2025) demonstrated that Thymus vulgaris extract enhances antioxidant capacity in chickens through multi-target mechanisms (Saci, 2025). A review by Sampath et al. (2025) indicated that the antioxidant effects of plant extracts (e.g., *Scutellaria baicalensis, Lonicera japonica*) are primarily associated with the activation of the Nrf2 signaling pathway (Sampath, 2025).

The antioxidant activity of RRP is mainly attributed to its rich phenolic compounds and polysaccharides. Huang et al. (2022) identified 59 phenolic compounds in RRP, with catechin, epicatechin, and isoquercitrin as the predominant flavonoids. In vitro assays confirmed that RRP polyphenols scavenge free radicals in a dose-dependent manner and reduce intracellular reactive oxygen species levels, while in vivo experiments in Caenorhabditis elegans demonstrated enhanced SOD and CAT activities (Huang, 2022). More importantly, Chen et al. (2023) further showed that bound polyphenols released from RRP dietary fiber exhibited potent antioxidant activity (Chen, 2023). These *in vitro* findings provide mechanistic support for the enhanced antioxidant enzyme activities and reduced MDA levels observed in the present study. While this was an antibacterial study, it powerfully illustrates the extract’s potent antioxidant mechanism, which in vivo could translate into direct free radical scavenging and activation of endogenous antioxidant enzyme systems. Research by Wang et al. (2022) also showed that *Rosa roxburghii Tratt.* polyphenol extract improves the oxidative stress status (Wang, 2022a). Furthermore, Huang et al. (2022) demonstrated that the extract reduces DNA damage, providing deeper evidence of its potent cytoprotective function (Huang, 2022).

### Effects of RRP on meat quality and chemical composition

Meat quality is a core indicator of broiler production efficiency. A key finding of this study is that RRP supplementation had no significant effect on physical meat quality traits (pH, drip loss, shear force, meat color). This indicates that RRP improves the nutritional profile of meat without compromising its sensory qualities, which is consistent with findings by Philatha et al. (2026) on olive pomace extract (Philatha, 2026). Regarding chemical composition, RRP significantly increased crude protein and decreased crude fat content in the breast muscle. This positive change may be linked to RRP's modulation of lipid and protein metabolism. Research on *Rosa roxburghii Tratt.* polyphenols has demonstrated significant hypolipidemic and anti-inflammatory effects (Wang, 2022a). Consistently, a study by Yang et al. (2025) demonstrated that *Rosa roxburghii Tratt.* polyphenols ameliorated colitis in mice, an effect associated with the regulation of gut microbiota and the upregulation of polyunsaturated fatty acid-derived metabolites (e.g., resolvin D2 and prostaglandin A1), further supporting the role of these bioactive compounds in modulating lipid metabolism (Yang, 2025a). Although the study by Chen et al. (2023) focused on antibacterial effects, its demonstration of the crude extract’s ability to disrupt microbial cell membranes indirectly reflects the potent biological activity of its active components (Chen, 2023). Given that membrane-associated processes are fundamental to host cell metabolism, such bioactivity may also extend to modulating host metabolic functions. More directly, a study on broilers by Islam et al. (2026) confirmed that plant extracts rich in bioactive components can improve carcass traits and dressing percentage (Islam, 2026). This positive change may be linked to RRP’s modulation of lipid and protein metabolism.

### Effects of RRP on fatty acid composition of breast muscle

Fatty acid composition is an important indicator of the nutritional value and health attributes of meat. In this study, RRP improved the fatty acid profile of the breast muscle, including increasing beneficial PUFA content, decreasing SFA content, improving the PUFA/SFA ratio, and lowering the ω−6/ω−3 ratio. These changes are beneficial to consumer health. Importantly, these beneficial changes are unlikely to be attributed to the direct fatty acid supply from RRP itself. As shown in Table 2, RRP contains only 1.58% crude fat; even at the highest inclusion level of 15%, this contributes merely 0.237% fat to the final diet, and at the recommended 10% level, the contribution is only 0.158%, a nutritionally negligible amount compared with the fat derived from soybean oil (3-4%) and corn (2-3%) in the basal diet. Therefore, the observed improvements in muscle fatty acid profile cannot be explained by exogenous fatty acid input from RRP, but rather reflect the modulatory effects of its bioactive components. The mechanisms by which RRP modulates fatty acid composition may involve the following. First, its potent antioxidant activity (as discussed in 4.2) can protect PUFAs from oxidative degradation, allowing them to accumulate in the muscle. Huang et al. (2024) directly demonstrated that *Rosa roxburghii Tratt.* extract enhances SOD activity and reduces ROS levels (Huang, 2024); Chen et al. (2023) showed its ability to disrupt biological membranes, an activity closely linked to its anti-lipid peroxidation properties (Chen, 2023). This in vitro evidence strongly supports the conclusion that RRP can protect unsaturated fatty acids in vivo. Second, the bioactive components in RRP may influence the expression of genes involved in lipid metabolism. Research by Wang et al. (2022) on *Rosa roxburghii Tratt.* polyphenols found they can regulate metabolites and gut microbiota associated with lipid metabolism (Wang, 2022b). The review by Sampath et al. (2025) also indicated that plant extracts can improve lipid metabolism in animals through various pathways (Sampath, 2025). The reduction in the ω−6/ω−3 ratio observed in this study further enhances the health value of the chicken meat, aligning with modern nutritional trends.

### Application value and future perspectives

This study systematically evaluated the potential of RRP as a functional feed ingredient in broiler production. The results indicate that a 100 g/kg inclusion level effectively promotes growth, enhances antioxidant capacity, and improves the nutritional composition of meat without compromising conventional meat quality. The 10% optimal level identified in this study is well within the range reported for other fruit pomaces in broiler nutrition, supporting its practical feasibility as a functional ingredient rather than a trace additive (Moroka, 2025). From a resource utilization perspective, converting RRP, a by-product of juice processing, into a high-value feed ingredient aligns with circular economy principles and offers a solution for agricultural waste management (Abdallah, 2019). From a production efficiency standpoint, RRP is widely available and inexpensive, providing strong potential for practical application. However, this study has limitations. First, the study by Han et al. (2022) on pesticide residues in *Rosa roxburghii Tratt.* reminds us that while focusing on the nutritional value, the safety of RRP as a processing by-product, such as the potential presence of pesticide or other contaminant residues, must be assessed before large-scale application (Han, 2022). Second, this study primarily evaluated the effects of RRP through phenotypic and biochemical parameters. The underlying molecular mechanisms, particularly its regulation of gut microbiota structure and signaling pathways such as Nrf2/ARE and NF-κB, require further investigation. Finally, the efficacy of RRP in different broiler strains and under various rearing conditions, as well as its economic feasibility, need further validation.

## Conclusion

This study tested the hypothesis that dietary supplementation with *Rosa roxburghii Tratt.* pomace (RRP) alleviates oxidative stress, thereby promoting growth and improving meat quality in broilers. The results confirmed that RRP supplementation at 100 g/kg, positioned as a functional feed ingredient rather than a conventional trace-level additive, effectively enhanced antioxidant capacity, improved growth performance and feed efficiency, and optimized the nutritional composition of breast muscle (increased protein and beneficial fatty acids, reduced fat) without compromising conventional meat quality traits. Higher supplementation at 150 g/kg conferred no additional benefit, establishing 100 g/kg as both the minimum effective dose and the optimal inclusion level within the tested range. The dose-response design, with gradients of 5%, 10%, and 15%, was essential to identify this plateau, as the 5% level proved insufficient to improve feed conversion ratio. In conclusion, dietary RRP supplementation at 100 g/kg is recommended as an effective, safe, and cost-effective strategy to improve broiler production performance and meat nutritional value. These findings provide a theoretical basis for the application of RRP as a functional feed ingredient and support the value-added utilization of agricultural by-products.

## Ethical approval and consent to participate

The animal experiments were approved by the Animal Welfare and Research Ethics Committee at Yangtze University (Jingzhou, China). All protocols and studies involving animals were conducted following the guiding principles of the Animal Care and Use Committee of Yangtze University (Jingzhou, Hubei, China, Approval No. 2025-166).

## Availability of data and materials

All data and materials are available. The data that support the findings of this study are available from the corresponding author upon reasonable request.

## CRediT authorship contribution statement

**Jing Zhang:** Writing – review & editing, Writing – original draft, Visualization, Validation, Supervision, Software, Resources, Project administration, Methodology, Investigation, Funding acquisition, Formal analysis, Data curation, Conceptualization. **Qiulin Xiao:** Writing – review & editing, Writing – original draft, Visualization, Validation, Supervision, Software, Resources, Project administration, Methodology, Investigation, Formal analysis, Data curation. **Liwei Guo:** Writing – review & editing, Writing – original draft, Visualization, Validation, Supervision, Software, Resources, Project administration, Methodology, Investigation, Formal analysis, Data curation. **Yunhe Wang:** Writing – review & editing, Validation, Resources, Investigation, Formal analysis, Data curation. **Wenqing Zhu:** Writing – review & editing, Validation, Investigation, Formal analysis, Data curation. **Xin Yin:** Writing – review & editing, Validation, Funding acquisition, Data curation. **Zhenglu Xie:** Writing – review & editing, Writing – original draft, Visualization, Validation, Supervision, Software, Resources, Project administration, Methodology, Investigation, Funding acquisition, Formal analysis, Data curation, Conceptualization.

## Disclosures

The authors declared that they have no conflicts of interest to this work.
